# Non-invasive pneumococcal pneumonia due to vaccine serotypes: A systematic review and meta-analysis

**DOI:** 10.1016/j.eclinm.2022.101271

**Published:** 2022-01-24

**Authors:** Louise Lansbury, Benjamin Lim, Tricia M McKeever, Hannah Lawrence, Wei Shen Lim

**Affiliations:** a Division of Epidemiology and Public Health, University of Nottingham, Nottingham, UK; b National Institute for Health Research (NIHR) Nottingham Biomedical Research Centre, UK; c Faculty of Biology (School of Medicine), University of Cambridge, Cambridge, UK; d Department of Respiratory Medicine, Nottingham University Hospitals NHS Trust, Nottingham, UK

**Keywords:** Pneumonia, *Streptococcus pneumoniae*, Pneumococcal vaccines, Serotype, Older adults

## Abstract

**Background:**

Non-invasive pneumococcal pneumonia causes significant morbidity and mortality in older adults. Understanding pneumococcal sero-epidemiology in adults ≥50 years is necessary to inform vaccination policies and the updating of pneumococcal vaccines.

**Methods:**

We conducted a systematic review and random-effects meta-analysis to determine the proportion of community-acquired pneumonia (CAP) in people ≥50 years due to pneumococcus and the proportion caused by pneumococcal vaccine serotypes. We searched MEDLINE, EMBASE and PubMed from 1 January 1990 to 30 March 2021. Heterogeneity was explored by subgroup analysis according to a) patient group (stratified versus age) and depth of testing, b) detection/serotyping method, and c) continent. The protocol is registered with PROSPERO (CRD42020192002).

**Findings:**

Twenty-eight studies were included (34,216 patients). In the period 1–5 years after introduction of childhood PCV10/13 immunisation, 18% of CAP cases (95% CI 13–24%) were attributable to pneumococcus, with 49% (43–54%) of pneumococcal CAP due to PCV13 serotypes. The estimated proportion of pneumococcal CAP was highest in one study that used 24-valent serotype-specific urinary-antigen detection (ss-UAD)(30% [28–31%]), followed by studies based on diagnostic serology (28% [24–33%]), PCR (26% [15–37%]), ss-UAD14 (17% [13–22%]), and culture alone (14% [10–19%]). A higher estimate was observed in Europe (26% [21–30%] than North America (11% [9–12%](*p*<0·001). PCV13-serotype estimates were also influenced by serotyping methods.

**Interpretation:**

Non-invasive pneumococcal CAP and vaccine-type pneumococcal CAP remains a burden in older adults despite widespread introduction of pneumococcal infant immunisation. Studies heavily reliant on ss-UADs restricted to vaccine-type serotypes may overestimate the proportion of potentially vaccine-preventable pneumococcal pneumonia. Sero-epidemiological data from low-income countries are lacking.

**Research in context**

**Evidence before this study**

We searched Medline, Embase and PubMed from 1 January 1990 to 30 March 2021, with no restriction by language, for studies evaluating the proportion of non-invasive pneumococcal pneumonia in adults aged 50 years and above caused by serotypes included in pneumococcal vaccines across the world. Observational studies have differed widely in the reported contribution of vaccine-type serotypes to non-invasive disease (from 25% to 80%), likely reflecting the use of different diagnostic and serotyping techniques, regional epidemiology and the impacts of national pneumococcal vaccination programmes. Our search did not reveal any previous systematic reviews that have assessed the impact of these factors on estimates of pneumococcal pneumonia and vaccine-preventable disease in older adults globally.

**Added value of this study**

This systematic review and meta-analysis, conducted at the request of the WHO Pneumococcal SAGE Working Group, is the first to focus on the impact of pneumococcal serotypes on non-invasive pneumococcal CAP in adults aged 50 years and above. We included 28 eligible studies (34,216 patients) from different parts of the world and have shown that pneumococcal serotypes included in current pneumococcal vaccines still cause a persistent burden of potentially vaccine preventable non-invasive CAP in older people despite the widespread introduction of infant immunisation with pneumococcal conjugate vaccines. Furthermore, we highlight the effect of pneumococcal diagnostic/serotyping method and geographical location on the estimates of pneumococcal and vaccine-type CAP, and identify an important evidence-gap through the absence of data from low-middle income countries (LMICs) where the burden from pneumonia remains very high.

**Implications of all the available evidence**

Strategies to further reduce the substantial proportion of vaccine-preventable non-invasive CAP in older people, despite infant pneumococcal immunisation programmes, warrant consideration. Studies from LMICs are required to guide global pneumococcal vaccination policies as there are currently no serotype specific data for non-invasive disease from these countries. Future studies should be supported by the development of improved serotype-specific diagnostic methods which detect a wide range of serotypes not restricted to those in existing vaccines so that changes in non-vaccine serotypes can be detected, and to reduce potentially biased estimates of vaccine-preventable disease.

Alt-text: Unlabelled box

## Introduction

Community-acquired pneumonia (CAP) is a significant cause of morbidity and mortality worldwide.^1^^,^^2^ *Streptococcus pneumoniae* (pneumococcus) is the most commonly implicated bacterial pathogen,^3^, ^4^, ^5^, ^6^, ^7^ and the spectrum of pneumococcal disease ranges from asymptomatic nasopharyngeal carriage through to localised infections and invasive pneumococcal disease (IPD), although non-invasive pneumococcal pneumonia is the most common manifestation.^8^ The Global Burden of Diseases Study estimated that in 2016 pneumococcal lower respiratory infections caused around 1·2 million deaths worldwide and 197 million episodes.^9^ The burden of pneumococcal disease follows a U-shaped curve, with greatest incidence and mortality in young children under 5 years and older adults aged 65 and above.^2^^,^^9^^,^^10^

With 100 documented pneumococcal serotypes, which vary in their disease severity, invasiveness and antimicrobial susceptibility, pneumococcal disease is at least partially vaccine-preventable. Many countries around the world have established infant immunisation programmes against pneumococcal disease, most using either a 13-valent or 10-valent pneumococcal conjugate vaccine (PCV). Introduction of childhood PCV immunisation has been associated with a reduction of overall and serotype-specific IPD in young children. Furthermore, infant PCV immunisation reduces nasopharyngeal carriage in those who are vaccinated thus preventing onward transmission to unvaccinated children and adults, and has resulted in reductions in both IPD and pneumococcal pneumonia across all ages.^11^, ^12^, ^13^, ^14^, ^15^ Some higher-income countries offer PPV23 and/or PCV13 vaccination to high-risk adults, including those over 65 years, although uptake varies between countries and is poor in many countries in which the vaccine is available.^16^^,^^17^

Most data on the epidemiology of vaccine-serotype pneumococcal disease is derived from invasive isolates, although fewer than 10% of pneumococcal pneumonia cases are invasive. To inform effective global pneumococcal vaccination strategies it is important to understand the true burden of non-invasive pneumonia and the impact of current vaccination programmes on the sero-epidemiology of the disease in older people. The primary objective of this systematic review and meta-analysis was to summarise the evidence on the proportion of non-invasive pneumococcal CAP due to *S. pneumoniae* serotypes covered by the PCV and PPV23 vaccines in people who are 50 years or older, and the secondary objective to evaluate the proportion of non-invasive pneumococcal CAP due to *S. pneumoniae*. This systematic review and meta-analysis was conducted at the request of the WHO Pneumococcal SAGE Working Group and initial results were presented to the Working Group in August 2020.

## Methods

We conducted this systematic review and meta-analysis in accordance with the Preferred Reporting Items for Systematic Reviews and Meta-Analyses (PRISMA) reporting guidelines.^18^ The study protocol was registered with the National Institute for Health Research international prospective register of systematic reviews (PROSPERO) (CRD42020192002).^19^

### Search strategy

We searched MEDLINE, EMBASE and PubMed from 1 January 1990 up to 30 March 2021, using search terms relating to community-acquired pneumonia, *Streptococcus pneumoniae,* and pneumococcal serotypes. (See Supplementary Material 1 for example of the EMBASE search strategy). We also manually searched the reference lists of included articles for relevant studies. No language restrictions were imposed.

### Study selection

We included cohort studies, surveillance studies and registry studies reporting data on non-invasive pneumococcal pneumonia caused by serotypes included in the study country's national pneumococcal immunisation schedule. Studies were eligible if they had separate pneumococcal data for adults aged 50 years or above, either stratified according to age group or in which the overall mean or median age of the entire cohort was ≥50 years, with a diagnosis of pneumonia acquired in the community. We excluded experimental studies, case-control studies, editorials, reviews and meta-analyses, studies with insufficient data on the pneumococcal serotypes causing non-invasive pneumonia in people aged 50 years and older, and studies which only included patients with invasive pneumococcal disease. One author (LL) screened the titles using pre-specified inclusion and exclusion criteria. Abstract and full text screening was independently performed by two authors (LL and HL/BL/TM) with disagreements resolved by consensus.

### Data extraction and analysis

Two reviewers (LL and HL/BL/TM) independently extracted data from individual studies using a predefined piloted template. We collected data on study methodology, location and setting, study population, national pneumococcal immunisation schedule including type and date of introduction, the incidence and proportion of CAP patients with pneumonia due to *S. pneumoniae* and the proportion of those due to vaccine serotypes, and the methods used to diagnose pneumococcal pneumonia and to identify pneumococcal serotypes. We assessed the risk of bias in the study group selection and outcome domains using a modification of the Newcastle-Ottawa Scale.^20^ The comparability domain was not considered relevant due to the design of the included studies.

### Outcome measures

Outcome measures were the proportion of CAP caused by *S. pneumoniae* in people aged 50 years and over, and the proportion of pneumococcal pneumonia in patients aged 50 and over in whom a vaccine type (VT) pneumococcus was identified as the causative pathogen. The serotypes covered by each of the currently licensed pneumococcal vaccines are listed in supplement 2.

### Data synthesis

We estimated the pooled proportion using the *metaprop* command in Stata and a random effects model.^21^ Variances were stabilised using the Freeman-Tukey double arcsine transformation which normalises the outcomes before pooling so that studies with proportions close to 0% or 100% were approximately estimated. For a study *i*, in which ri denotes the number of observations with a certain characteristic and ni is the total number of observations, this is defined as:

$$\sin^{-1}\sqrt{\frac{ri}{ni+1}\;}+\;\sin^{-1}\sqrt{\frac{ri+1}{ni+1}}$$

The asymptotic variance of the transformed variable is defined as:

$$\frac{1}{ni+0.5}$$

The DerSimonian Laird method was used to compute pooled estimates based on the transformed values and their variances.

We assessed heterogeneity using the I^2^ statistic.

We estimated the proportion of a) patients aged 50 and above with CAP caused by pneumococcus; b) patients aged 50 and above with pneumococcal pneumonia caused by a serotype covered by PCV13 and PPV23. Analyses were stratified by: patient group (entire cohort aged ≥50 years or median/mean ≥50 years) and completeness of testing (with optimal testing defined as simultaneous testing by culture, BinaxNOW (non-specific pneumococcal antigen test) and serotype-specific urinary antigen detection (ss-UAD)/serotype specific serology); by serotype detection method (UAD24, UAD14, serology, PCR, respiratory culture only); and by continent (Asia, Europe and North America). Data were analysed for the periods pre- and post-introduction of childhood PCV10/13 immunisation programmes, with data for the post-PCV 10/13 period being included if collected at least one year after national introduction of PCV10/13.

All analyses were conducted in Stata 16·0 software (StataCorp. 2019. Stata Statistical Software: Release 16. College Station, TX:StatCorp LLC.).

### Ethics statement

Ethical approval for this systematic review and meta-analysis is not applicable since the data utilised were collected from previously published research in the literature. All the included studies in this review had received ethical approval prior to data collection.

### Role of the funding source

The funders had no role in the study design, data collection, data analysis, data interpretation, or in the writing of the manuscript.

## Results

**Figure 1:**
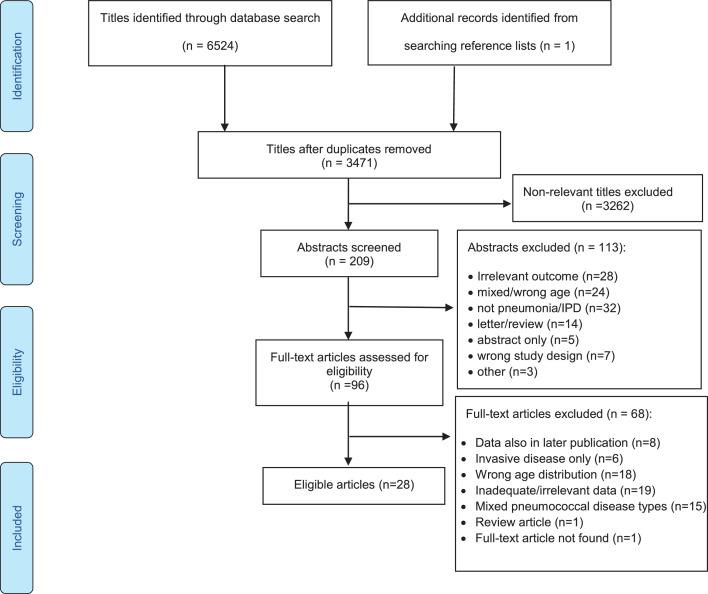
PRISMA flow diagram for study selection.

After screening 3471 papers, 28 studies were eligible for inclusion (Figure 1)^15^^,^^22^, ^23^, ^24^, ^25^, ^26^, ^27^, ^28^, ^29^, ^30^, ^31^, ^32^, ^33^, ^34^, ^35^, ^36^, ^37^, ^38^, ^39^, ^40^, ^41^, ^42^, ^43^, ^44^, ^45^, ^46^, ^47^, ^48^ and included data on 34,216 patients from 19 countries across Asia, Europe and North America (supplementary Table 1). All studies were from high income countries. The most common reasons for exclusion of studies were wrong age group and inadequacy of serotype data.

In the modified risk of bias assessment, 8 of the 28 (28·6%) studies were judged to have some degree of selection bias as included patients may have not been truly representative of patients with CAP (Supplementary Table 2).

### Proportion of CAP due to *Streptococcus pneumoniae*

**Figure 2:**
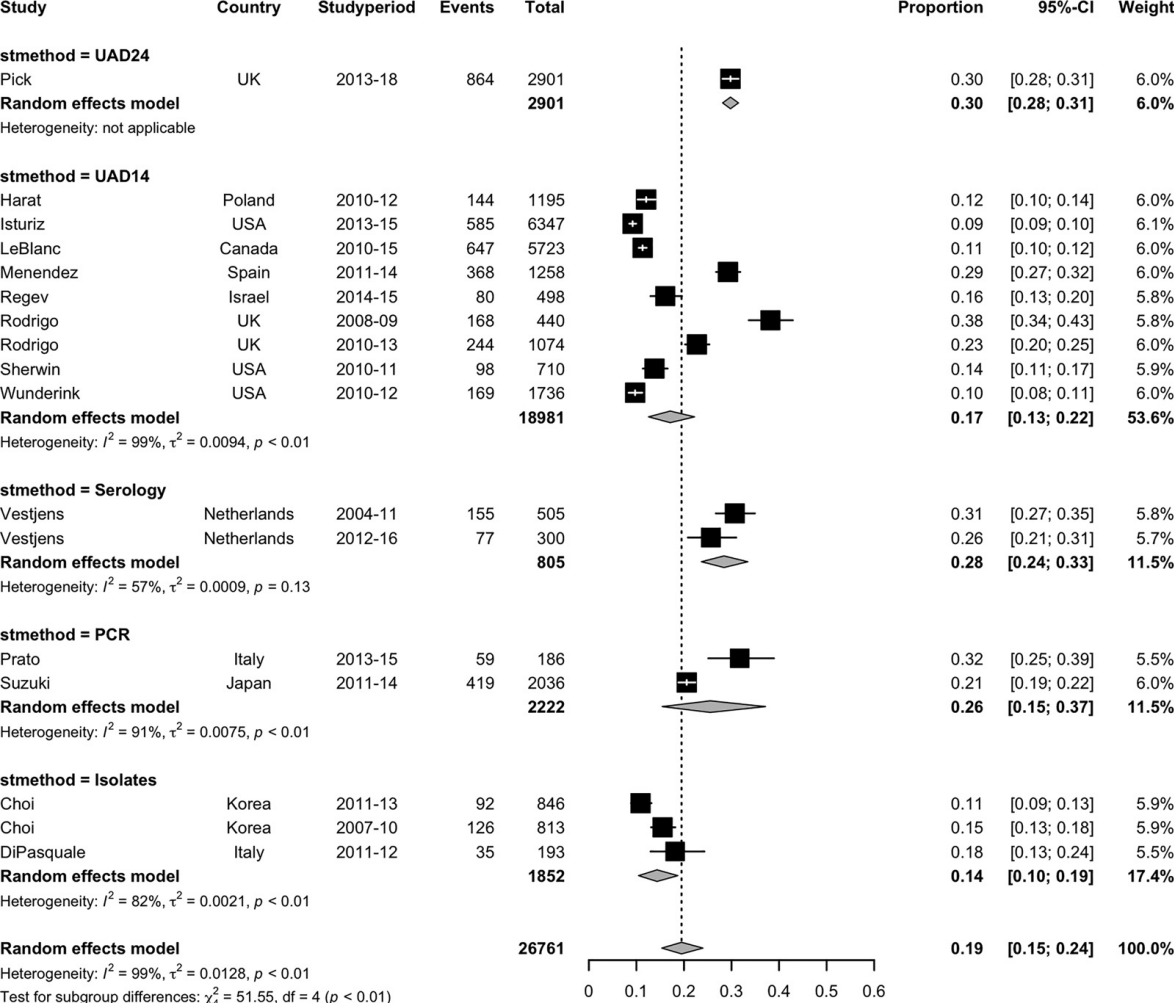
Forest plot of the pooled estimated proportion of CAP due to *Streptococcus pneumoniae*, stratified by the method of pneumococcal serotyping employed by included studies. Key: UAD24: 24-valent serotype-specific urinary antigen detection, UAD14: 14-valent serotype-specific urinary antigen detection, PCR: polymerase chain reaction (*S. pneumoniae* isolated by PCR with sequential multiplex PCR using 29 serotype specific primer pairs). Sensitivity analysis excluding 1 study (di Pasquale) considered to be at risk of some selection bias: UAD24: 30% (28–31), UAD14: 17% (13–22), Serology: 28% (24–33), PCR: 26% (15–37), Isolates: 13% (11–15), Overall I^2^=98.75%.

Prior to introduction of childhood PCV10/13 immunisation, the pooled estimated proportion of CAP which was due to pneumococcus in people aged 50 years and above was 22% (95% CI 15–29%, 6 studies, I^2^ = 97·1%),^15^^,^^25^^,^^27^^,^^42^^,^^44^^,^^46^ ranging from 12% to 38%. (Supplementary Figure 1) Eleven studies included data from at least one year after the introduction of PCV10/13, with a pooled proportion of 18% (95% CI 13–24%, I^2^= 98·9%), ranging from 9% to 32%.^15^^,^^22^^,^^24^^,^^25^^,^^27^^,^^30^^,^^31^^,^^33^^,^^37^^,^^39^^,^^41^ The estimated proportion of CAP due to pneumococcus was influenced by the testing method, with stratification according to pneumococcal detection method showing significant subgroup heterogeneity (*p*<0·001). The highest estimated proportion of CAP due to pneumococcus was seen with ss-UAD24 (30% [95% CI 28–31%], 1 study), followed by serology (28% [95% CI 24–33%], 2 datasets), PCR (26% [95% CI 15–37%], 2 studies), ss-UAD14 (17% [95% CI 13–22%, 9 datasets), and culture alone (14% [95% CI 10–19%], 3 datasets). (Figure 2)

**Figure 3:**
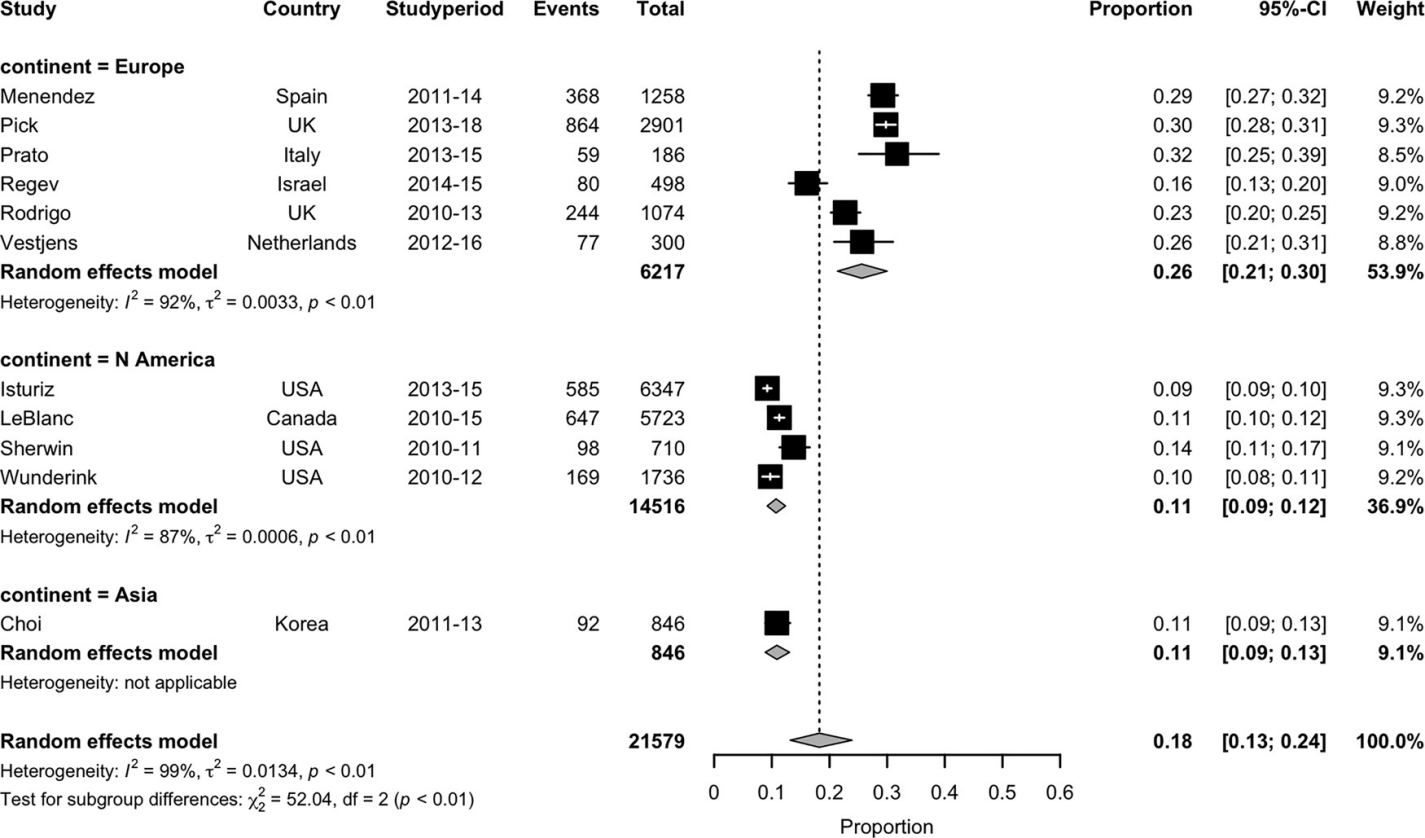
Estimated proportion CAP due to *S. pneumoniae* stratified by continent.

Significant heterogeneity was noted between studies stratified by continent (*p*<0·001)(Figure 3). In the post PCV10/13 period, the summary estimate for the proportion of CAP due to pneumococcus from six studies in Europe was 26% (95% CI 21–30%, I^2^=92%),^15^^,^^24^^,^^25^^,^^30^^,^^33^^,^^41^ whereas from four North American studies it was 11% (95% CI 9–12%, I^2^=87·2%).^22^^,^^31^^,^^37^^,^^39^ Similarly, one study from Korea also reported a proportion of 11% (95% CI 9–13%), although this was based on serotyped isolates from bacterial culture rather than ss-UAD testing so may be an underestimate of the true proportion.^27^

No significant subgroup differences were observed when studies were grouped by definition of age groups (defined strata versus mean/median age) (*p* = 0·64), nor by depth of testing (optimal versus sub-optimal)(*p* = 0·71)(Supplementary Figures 2 and 3).

### Proportion of pneumococcal CAP due to PCV13 serotypes

The overall pooled proportion of pneumococcal CAP which was due to PCV13 serotypes in the 1–5 years post PCV10/13 period was 49% (95% CI 43–54%, 15 studies, 19 datasets, I^2^=92·7%)(Supplementary Figure 4). For studies in which pneumococcal testing was optimal and which stratified by age, the pooled proportion of pneumococcal CAP due to PCV13 serotypes was similar to the overall estimate, 51% (95% CI 43–59%, 7 studies, I^2^=92·7%).^15^^,^^22^^,^^30^^,^^31^^,^^37^, ^38^, ^39^^,^^41^ The pooled proportion of all CAP due to PCV13 serotypes from these same studies was 8% (95% CI 6–11%, I^2^=97·1%).

**Figure 4:**
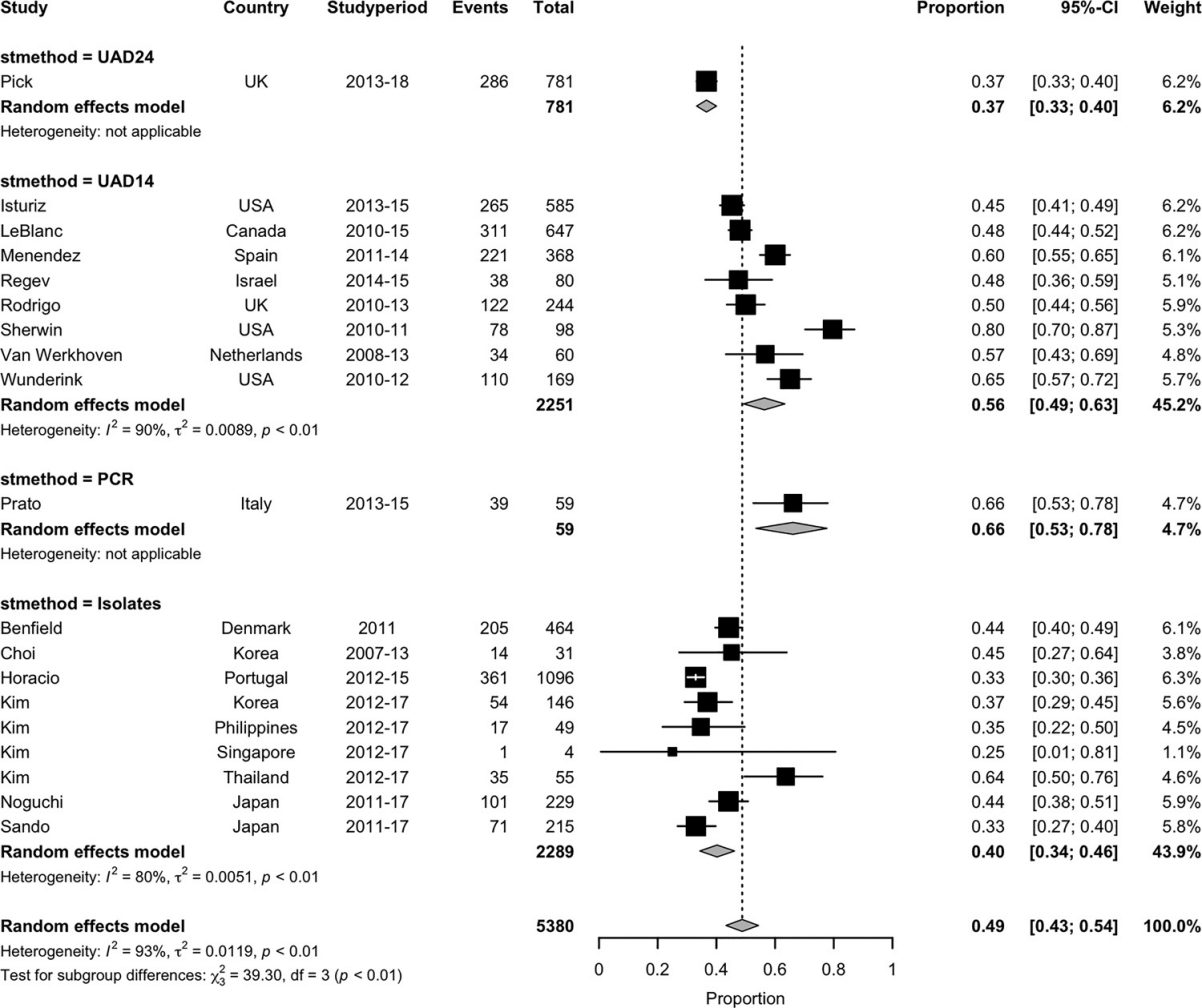
Estimated proportion of pneumococcal CAP due to PCV13 vaccine serotypes in the period after introduction of PCV10/13 immunisation programmes, stratified by the method of pneumococcal serotyping employed by included studies. Legend: Sensitivity analysis excluding 4 studies (Benfield, Horacio, Kim, Sando) considered to be at risk of some selection bias: UAD24: 37% (33–40), UAD14: 56% (49–63), PCR: 66% (53–77), Isolates: 54% (47–60), Overall I^2^=92.14%.

Again, the estimates were influenced by the method of pneumococcal serotype detection (Figure 4). The highest estimated proportion of pneumococcal CAP due to PCV13 serotypes was observed in one study in which sequential-multiplex PCR was used for serotyping using 29 serotype-specific primer pairs (66%, 95% CI 53–78%).^33^ The estimated pooled proportion of pneumococcal CAP due to PCV13 serotypes from studies employing a 14-valent ss-UAD was 56% (95% CI 49–63%, 8 studies, I^2^=90·2%),^15^^,^^22^^,^^24^^,^^30^^,^^31^^,^^37^, ^38^, ^39^ and 40% (95% CI 34–46%, I^2^= 80·4%) from six studies in which serotyping was performed on isolates only.^26^, ^27^, ^28^, ^29^^,^^35^^,^^43^ In the one study which utilised a 24-valent ss-UAD, the proportion of pneumococcal CAP due to PCV-13 serotypes was 37% (95% CI 33–40%).^41^

By continent, the highest proportion of pneumococcal CAP caused by PCV13 serotypes was noted when studies from North America were pooled, all of which employed ss-UAD14 (59%, 95% CI 47–71%, 4 studies, I^2^=95%),^22^^,^^31^^,^^37^^,^^39^ followed by Europe (48%, 95% CI 40–56%, 8 studies, I^2^=94·3%) for which serotyping methods varied between the included studies.^15^^,^^24^^,^^28^^,^^30^^,^^33^^,^^38^^,^^41^^,^^43^ The pooled estimate was lowest in studies from Asia, all of which were based on serotyping of pneumococcal isolates (41%, 95% CI 34–49%, 4 studies, I^2^=70·1%).^26,27,29,35^(Supplementary Figure 5)

### Proportion of pneumococcal CAP due to PPV23 serotypes

The overall pooled estimate of the proportion of pneumococcal CAP due to PPV23 serotypes, including those in PCV13, was 67% (95% CI 62–72%, 12 studies, I^2^=90·3%). Two studies which used PCR for serotyping had a pooled estimated proportion of 80% (95% CI 75–83%). One of these studies examined for 50 pneumococcal serotypes by nanofluidic real-time PCR and the second employed 29 primer pairs to serotype pneumococcus PCR-positive samples.^33^^,^^46^ The proportion of CAP due to PPV23 serotypes in the one study which used SS-UAD24 to detect pneumococci was 70% (95% CI 66–73%).^41^ Pooling data from the nine studies which serotyped using the Quellung method on pneumococcal isolates only gave the lowest estimated proportion of CAP due to PPV23 serotypes at 65% (95% CI 58–71%, I^2^=88·2%).^26–29,32,35,40,43,45^(Supplementary Figure 6)

### Prevalent pneumococcal serotypes in CAP

The most prevalent pneumococcal serotypes for each study are presented in Table 1. Overall serotype 3 ranked as one of the top three most prevalent serotypes in 25 of 30 (83·3%) datasets for non-bacteraemic CAP, followed by 19A (53·3% of datasets), and 11A/E and 19F (both 30% of datasets).

**Table 1:** Pneumococcal serotypes with the highest prevalence causing CAP in the included studies.

Study	Country	Year	Pre PCV^a^/Years post PCV infant immunisation	Number of people included	Most prevalent serotypes
**Europe**					
Domenech^45^	Spain	2001–8	Pre	255	3, 1, 5 (bacteraemic) 3, 19F, 23F (non-bacteraemic)
Harat^42^	Poland	2010–12	Pre	1195	3, 23F, 18C
Pletz^34^	Germany	2002–11	Pre	391	7F, 3, 1
Vila Corcoles^40^	Spain	2008–11	Pre	125	3, 19A, 22F
Horacio^36^	Portugal	1999–2011	Pre to 1	225	3, 11A, 19F
Benfield^43^	Denmark	2011	1	464	3, 11A,19A (non-bacteraemic) 1, 7F, 3 (bacteraemic)
Van Werkhoven^38^	Netherlands	2011–13	Up to 2	288	3, 7F, 19A
Rodrigo^15^	UK	2008–13	Up to 3	1887	1, 7F/A, 19A
Menendez^24^	Spain	2011–14	1–3	1258	3, 7F, 19A
Di Pasquale^44^	Italy	2011–12	2–3	193	35F, 3, 24
Regev^30^	Israel	2014–15	4	498	3, 23F, 19A
Vestjens^25^	Netherlands	2012–16	1–5	805	3, 8, 19A
Horacio^28^	Portugal	2012–15	2–5	1096	3, 11A, 19F
Prato^33^	Italy	2013–15	3–5	186	23F, 9 V, 19A
Quirk^48^	Iceland	2009–17	Pre, up to 6	430	19F, 6B, 3 19F, 3, 11A
Pick^41^	UK	2013–18	3–8	2901	3, 8, 15A
**North America**					
Sherwin^31^	USA	2010–11	1	710	19A, 7F/A, 3 and 5
Wunderink^37^	USA	2010–12	Up to 2	2736	19A, 3, 7F
LeBlanc^22^^,^^47^	Canada	2011–15	1–5	5723	3, 7F, 19A
Isturiz^39^	USA	2013–16	3–5	6347	19A, 3, 5
**Asia**					
Oishi^32^	Japan	2001–03	Pre	114	19F, 23F, 6B
Morimoto^23^	Japan	2011–13	Pre	1772	3, 11A, 19F
Choi^27^	Korea	2007–13	Pre, up to 3	2221	3, 19A, 11A/E 3, 19A, 6C
Kim^26^	China	2008–14	Pre	194	19F, 19A, 23F
	Korea	2008–14	2–5	146	11A, 19A, 19F
	Philippines	2008–14	2–5	49	3, 11A, 6B/19A
	Thailand	2008–14	1–4	55	3, 23F, 6B
Suzuki^46^	Japan	2011–14	Up to 1	2036	3, 10A, 6A/B
Sando^35^	Japan	2011–14, 2016–17	Pre, 3	438	3, 11A, 19 35B, 3, 6C/19A
Noguchi^29^	Japan	2011–17	Up to 4	229	3, 19F, 11A/E

Notes:

a PCV10 or PCV13; (pre) – pre-PCV 10/13 in infant immunisation programme; (post) – post introduction of PCV 10/13 in infant immunisation. Of the serotypes listed in the table: PCV7 serotypes – 6B, 9 V, 18C, 19F, 23F; PCV13Non7 serotypes – 1,3,5,6C, 7F, 19A; PPV23NonPCV13 serotypes – 8, 22F, 10A, 11A; Non-vaccine serotypes – 7A, 15A, 24, 35B, 35F.

### Trends in vaccine-type CAP post PCV10/13 infant immunisation

Overall trends in vaccine serotype and non-vaccine serotype pneumococcal pneumonia are described in Table 2.

**Table 2:** Trends in vaccine serotype and non-vaccine serotype pneumococcal pneumonia after the introduction of infant PCV immunisation.

Pneumococcal Serotype	Temporal trend in serotypes causing pneumococcal CAP	Studies reporting
**PCV7**	Overall decrease	○ Rodrigo^15^: 88% decrease pre to post PCV13 period (IRR/year 0·84 (95% CI 0·8–0·89, *p*<0·001)) ○ Sando^35^: Difference in hospitalised PCV7 ST pneumonia pre to 3 years post = −11% (95% CI −17·7 to −4·2, *p* = 0·02) ○ Vestjens^25^: decrease between pre-PCV10 period up to 2 years post (OR 0·19 (95% CI 0·05–0·76)) ○ Van Werkhoven^38^: decrease from 28% pre PCV10 to 0·7% 2 years post (p-value for trend 0·048) ○ Noguchi^29^: downward trend from 46·4% in 2011 to 4·3% in 2014 when PCV13 licensed for adults ≥65 years
	Persistence	○ Sherwin^31^: persistence of PCV7 STs 1 year after introduction of PCV13 into paediatric population
**PCV13Non7**	Overall decrease	○ Rodrigo^15^: 30% decrease pre to post PCV13 period (IRR/year 0·70 (95% CI 0·51–0·96, *p* = 0·024), but non-significant increase in STs 6B and 19F in final year ○ Sando^35^: Difference in hospitalised PCV13non7 ST pneumonia pre to 3 years post = −9% (95% CI −17·4 to −0·7, *p* = 0·035) ○ Wunderink^37^: non-significant decline up to 2 years post PCV introduction (adjusted OR 0·65 (95% CI 0·4–1·04)
	Persistence	○ Sherwin^31^: persistence of PCV13Non7 STs 1 year after introduction of PCV13 into paediatric population
**PCV13**	Overall decrease	○ Horacio^28^: decrease from 44% in year of PCV13 introduction to 29·7% after 5 years *p*<0·001) with decline in STs 3 and 19 ○ Sando^35^: decrease in hospitalised PCV13 ST pneumonia from 53% to 33% (*p*<0·001) pre to 3 years post PCV introduction ○ Choi^27^: Non-significant trend towards decrease in first 2 years after introduction (*p* = 0·062) ○ Noguchi^29^: downward trend from 71·4% in 2011 to 33·3% in 2015, but no significant change up to 3 years after PCV13 licensed for adults ≥65 years ○ Isturiz^39^: Decline from 5·1% to 3·4% over 3 year study period up to 5 years after childhood PCV and 3 years after ACIP recommendation of PCV13 in adults ≥65 years (*p* = 0·01). Reduction in all PCV13 STs except ST3 ○ Menendez^24^: Non-significant decreasing trend from 67·4% to 52·1% (*p* = 0·057) from 1 to 3 years after infant PCV13 introduction ○ LeBlan:c^22^ Variable – highest incidence of PCV13 STs in first 3 years after PCV13 introduction for infants, lowest in year 4 with significant decline in 50–64 year age group (*p* = 0·032]. Significant declines in STs 7F in ≥65 years and ≥50 years during first 3 years (*p* = 0·048 and 0·002 respectively) and ST3 in ≥65 years 9p=0·018) although incidence of ST3 increased again 4–5 years after infant PCV introduction in both in ≥65 years and ≥50 years (*p*<0·001 and *p* = 0·001 respectively).
**PCV10**	Overall decrease	○ Quirk^48^: 403·2 to 61·7/100,000 (*p*<0·001) up to 6 years after introduction, significant decline in STs 6B, 19F, 23F
**PCV10Non7**	No change	○ Vestjens^25^: no significant change up to 5 years after PCV10 replaced PCV7 (RR 1·08 (95% CI 0·3 – 3·86) ○ Van Werkhoven^38^: no significant change pre and up to 2 years post PCV10
**Non-vaccine serotypes**	Overall increase	○ Horacio^28^: increase from 27·0 to 41·9 between 2010 and 2015 (*p*<0·001) ○ Van Werkhoven^38^: increase in non-PCV13 STs from 30% pre to 37% in post PCV period (p-value for trend 0·048)
	No change	○ Quirk^48^: no significant change pre PCV10 introduction up to 6 years post

## Discussion

Our systematic review and meta-analysis indicates that in people ≥50 years, the proportion of CAP due to pneumococcus has remained substantial even after the introduction of infant immunisation programmes using PCV vaccines, ranging from 9% up to 30% in the included studies. Overall, almost 50% of serotypes causing pneumococcal CAP were covered by the PCV13, ranging from 25% to 80%, and 67% by PPV23, ranging from 50% to 82% in individual studies.

Of the factors influencing the results, the type of testing employed for pneumococcal detection and serotyping had a significant impact on the estimated proportions with lower estimates from those studies in which conventional techniques only were employed compared to newer methods such as ss-UAD and PCR. Serotype-specific multiplex urinary immunoassays have been developed which detect specific pneumococcal serotypes covered by PCV13 and/or PPV23.^49^, ^50^, ^51^ These ss-UADs detect additional pneumococcal cases of CAP that would otherwise be missed by traditional culture or BinaxNOW alone.^37^^,^^39^ These non-cultural tests (ss-UAD or PCR) have a higher sensitivity for the detection of pneumococcal infection compared to conventional culture with serotyping of isolates using the Quellung reaction. Consequently, studies that rely heavily on ss-UADs for the detection of pneumococcal cases will over-estimate the proportion of pneumococcal CAP due to the serotypes detected by the ss-UADs used; for ss-UAD14 and ss-UAD24 these correspond largely to the VTs in PCV13 and PPV23 respectively. It should also be considered that the higher sensitivity of ss-UADs and PCR tests may be detecting pneumococcal carriage and not true lower respiratory tract infection. Conversely, studies using traditional detection methods such as blood and/or sputum culture alone are likely to underestimate the true burden of pneumococcus. The diagnostic sensitivity of sputum culture is highly variable and will depend upon the quality of the sample, processing delays and prior antimicrobial therapy, and false positives from upper respiratory tract carriage may occur.^52^

Detection of pneumococcal C-polysaccharide antigen by the BinaxNOW urinary antigen test has been estimated to have increased diagnosis of pneumococcal CAP by 11–23% beyond conventional culture.^53^^,^^54^ It has an estimated sensitivity and specificity of 75% and 95% respectively,^53^, ^54^, ^55^, ^56^ although several studies found that sensitivity is higher for bacteraemic than non-bacteraemic pneumonia (77–92% and 52–78% respectively).^57^, ^58^, ^59^, ^60^ Sensitivity has also been shown to vary according to pneumococcal serotype and temporal changes in sensitivity have been associated with changes in the distribution of serotypes with time, sensitivity being highest for serotypes 9 V, 14, 18C and 20, and lowest for STs 8, 9 L/N, 11A, 23B and non-typeable serotypes.^61^

We observed that the proportion of CAP due to pneumococcus varied according to geographical region, with a pooled estimate of 26% from European studies compared to 11% in North America. This is in accordance with other studies which have found similar discrepancies between continents.^62^, ^63^, ^64^ This observation may represent a true difference in the proportion of CAP caused by pneumococcus and an increase in non-vaccine serotypes in both invasive and non-invasive pneumococcal disease in Europe which has not been seen in North America, or it may be due to the use of the 24-valent ss-UAD in the UK and thus a reflection of the types of tests employed. Other factors which may partially explain the finding include differences in the prevalence of tobacco smoking, in pneumococcal vaccine coverage, disparities between healthcare systems and access to antibiotics.^65^^,^^66^^,^^67^

It was notable in our review that none of the data on VT non-invasive pneumococcal pneumonia were from LMICs. Currently only invasive pneumococci are serotyped in most sub-Saharan African countries. One recent study from Malawi using the non-serotype specific BinaxNOW assay suggested that, following introduction of universal infant PCV in 2011, pneumococcal CAP still accounted for 21·4% of pneumonias in hospitalised adults. No data on serotype distribution has been described.^68^ A South African study confirmed that ss-UAD has high diagnostic accuracy to detect pneumococcal pneumonia and simultaneous detection of PCV13 serotypes in HIV-infected adults in South Africa, offering a potentially useful tool to determine pneumococcal pneumonia in this population.^69^

Of note is the persistence of ST3 as one of the most prevalent vaccine serotypes both before and after the PCV13 infant programme. A recent global genomic analysis of ST3 has identified a new antibiotic-resistant clade which is replacing the less resistant clade.^70^ Multiple reports suggest that the indirect protective effects of infant PCV immunisation against IPD in adults is less for ST3 than for other PCV serotypes.^12^^,^^71^, ^72^, ^73^, ^74^, ^75^ It has been reported that PCV13 is less effective at decreasing ST3 colonisation in children, and therefore may be insufficient to indirectly protect older adults.^76^

Our systematic review and meta-analysis has some limitations in addition to those already discussed. Our search strategy sought to identify studies with quantitative data on pneumococcal serotypes, so studies not reporting on serotypes would not have been included in our analysis of the overall proportion of CAP caused by *S. pneumoniae*. Sampling methods within populations were not standardised across studies and varied from convenience sampling to recruitment of all eligible patients in a cohort and this will affect the overall estimates of proportions. Although we focused on community-acquired infections, we cannot exclude the possibility that some included patients may have had non-community acquired infections. The true incidence of vaccine-type pneumococcal pneumonia is likely to be underestimated from studies which only included patients hospitalised with CAP; there is evidence from several studies that many cases of CAP are not hospitalised and the percentage treated as outpatients or in primary care will vary depend upon the different set up of healthcare systems in different countries.^5^^,^^6^^,^^77^ Some countries have also introduced adult pneumococcal vaccination policies which may affect variations in the incidence of pneumococcal disease; recommended risk groups may differ between those countries and coverage is likely to vary substantially. As most data were only collected for the period up to five years following the introduction of childhood PCV10/13 immunisation, this may not have been sufficient to allow for the development of herd protection.

Based on the findings of the systematic review and meta-analysis we make the following recommendations. First, data on the contribution of pneumococcal serotypes to non-invasive CAP in LMICs are sorely needed to better guide pneumococcal vaccine policy in these regions and tailored vaccine development. The incidence of pneumococcal infection in LMICs is high and pneumococcal vaccines, particularly PCVs, are expensive compared to other vaccines, yet it is not certain that the serotypes included in the current pneumococcal vaccines match the prevailing serotypes causing pneumococcal pneumonia in LMICs. Second, more studies are required to strengthen the evidence on the sero-epidemiology of CAP in older people, as the majority of pneumococcal infections in adults aged ≥50 years are non-invasive yet most of the data come from IPD which is much less common and may be caused by serotypes which do not reflect the pattern of serotypes causing non-invasive pneumonia. Integral to this is the need to develop better serotype-specific diagnostic methods capable of detecting a wider range of serotypes and not restricted to VTs in the existing vaccines so that trends in non-vaccine serotypes can also be detected. This will also be important as new pneumococcal vaccines emerge which cover more serotypes, such as PCVs which are 21-valent. New diagnostics should not rely on respiratory samples, such as sputum PCR, as 30 to 40% of adults with pneumonia do not produce sputum and such specimens are prone to contamination or may only reflect carriage.^52^^,^^78^, ^79^, ^80^ Third, we highlight caution when interpreting studies heavily reliant on ss-UADs that target only VT serotypes as they may bias towards over-estimates of the proportions of potentially vaccine-preventable disease.

In conclusion, this review of pneumococcal CAP in adults aged 50 years and over highlights variations in the types of studies and methodologies used to determine the burden of pneumococcal vaccine serotypes. Nonetheless, it demonstrates that there remains a considerable burden of pneumococcal CAP, including potentially vaccine preventable disease, in older people.

## Declaration of interests

WSL reports unrestricted investigator-initiated research funding from Pfizer from 2016 to present for an unrelated multi-centre study in pneumonia in which he is the CI, and research funding for unrelated clinical trials in the fields of COVID-19, tuberculosis and community-acquired pneumonia. WSL also declares unpaid roles as the Joint Committee on Vaccination and Immunisation (JCVI) UK Chair of COVID-19 Immunisation, and National Lead of the British Thoracic Society community acquired pneumonia audit programme. LL's salary is funded by the National Institute for Health Research (NIHR) Nottingham Biomedical Research Centre, UK. HL declares voluntary, unpaid membership of the National Confidential Enquiry into Patient Outcome and Death specialist advisory group for Community Acquired Pneumonia. TM and BL declare no competing interests.

## Appendix Supplementary materials

Image, application 1
