# Supplementary material for: Non-invasive pneumococcal pneumonia due to vaccine serotypes: A systematic review and meta-analysis
Source: eClinicalMedicine. 2022 Jan 24;44:101271. doi: 10.1016/j.eclinm.2022.101271 (PMC8790487; doi:10.1016/j.eclinm.2022.101271)
Supplement: Supplementary file 1 [file mmc1.docx]

**SUPPLEMENTARY MATERIALS**

**Non-invasive pneumococcal pneumonia due to vaccine serotypes: a systematic review and meta-analysis**

**Contents**

[Supplementary Material 1: Example of Search Strategy for EMBASE 2](#_Toc90291009)

[Supplementary Material 2: Serotypes included in pneumococcal vaccines 2](#_Toc90291010)

[Supplementary Table 1: Characteristics of included studies 3](#_Toc90291011)

[Supplemental Table 2: Modified Newcastle-Ottawa Scale risk of bias assessment 8](#_Toc90291012)

[Supplementary Figure 1: Estimated proportion of CAP due to *S.pneumoniae* in the periods prior to and post introduction of PCV10/13 immunisation in children 9](#_Toc90291013)

[Supplemental Figure 2 : Estimated proportion of CAP due to *S.pneumoniae* stratified by definition of age 10](#_Toc90291014)

[Supplemental Figure 3: Proportion of CAP due to *S.pneumoniae* stratified by depth of testing 11](#_Toc90291015)

[11](#_Toc90291016)

[Supplemental Figure 4: Estimated proportion of pneumococcal CAP caused by PCV13 vaccine type *S. pneumoniae* in the period after the introduction of PCV 10/13 programmes 12](#_Toc90291017)

[Supplemental Figure 5: Estimated proportion of pneumococcal CAP due to PCV13 vaccine serotypes – stratified by continent. 13](#_Toc90291018)

[Supplemental Figure 6: Estimated proportion of pneumococcal CAP due to PPV23 vaccine type stratified by serological test method 14](#_Toc90291019)

## Supplementary Material 1: Example of Search Strategy for EMBASE

1. pneumonia/ or Streptococcus pneumonia/ or bacterial pneumonia/ or community acquired pneumonia/

2. (strep* pneumo* or S pneumo* or pneumococc*).mp. [mp=title, abstract, heading word, drug trade name, original title, device manufacturer, drug manufacturer, device trade name, keyword, floating subheading word, candidate term word]

3. 1 or 2

4. (serotype or serogroup).mp. [mp=title, abstract, heading word, drug trade name, original title, device manufacturer, drug manufacturer, device trade name, keyword, floating subheading word, candidate term word]

5. 3 and 4

6. (incidence or prevalence or distribution or frequency).mp. [mp=title, abstract, heading word, drug trade name, original title, device manufacturer, drug manufacturer, device trade name, keyword, floating subheading word, candidate term word]

7. 5 and 6

8. limit 7 to (human and yr="1990 -Current")

The MeSH thesaurus headings and free-text terms above were adapted to search MEDLINE and PubMed.

## Supplementary Material 2: Serotypes included in pneumococcal vaccines

| Vaccine type | SPn serotypes included |
| --- | --- |
| PCV7 | 1, 5, 6B, 14, 18C, 19F, 23F |
| PCV10 | 1, 4, 5, 6B, 7F, 9V 14, 18C, 19F, 23F |
| PCV13 | 1, 3, 4, 5, 6A, 6B, 7F, 9V, 14, 18C, 19A, 19F, 23F |
| PPV23 | 1, 2, 3, 4, 5, 6B, 7F, 8, 9N, 9V, 10A, 11A, 12F, 14, 15B, 17F,18C, 19A, 19F, 20, 22F, 23F, 33F |

## Supplementary Table 1: Characteristics of included studies

| **Study** | **Country** | **Design** | **Study period** | **Lab identification of SPn and serotype detection method** | **Age** | **SPn pneumonia/total CAP (%)** | **Pre PCV and/or time post PCV 10/13 introduction (years)** | **VT pneumonia/total SPn pneumonia (%) post introduction of PCV programme** | **Included in MA*** |
| --- | --- | --- | --- | --- | --- | --- | --- | --- | --- |
|  |  |  |  |  |  |  |  |  |  |
| Benfield 2013 | Denmark | Population-based, consecutive first episodes SPn pneumonia | January-December 2011 | SPn cultured from LRT and/or blood; pneumotest latex and Quellung reaction | Median 68 (IQR 58-78) | n/a | 1 | **Non-bacteraemic:**  PCV13: 91/272 (33.4)  PPV23: 155/272 (57.0)  **Bacteraemic:**  PCV13: 114/192 (59.4)  PPV23: 169/192 (88.0) | **f,g,h,i** |
| Choi 2015 | Korea | RC | 2007-2013 | Culture (blood, pleural effusion, adequate LRT) or BinaxNOW | Mean 65.5 | 276/2221 (12.6) | 2-3 | PCV13: 14/31 (45.2)  PPV23: 17/31 (54.8) | a,b,c,d,e,f,g,h,i |
| Di Pasquale 2017 | Italy | PC | October 2011-October 2012 | Blood, sputum and/or NP swab, tracheal aspirate, pleural fluid, urinary antigens. PCR of isolates | 78 (66-84) | 35/193 (18) | 2-3 | ‘about 50%’ | a,b,d,e |
| Domenech 2011 | Spain | P, lab-based, COPD patients only | 2001-2008 | Blood culture/respiratory specimen. Quellung reaction, multiplex PCR | Mean 70 ±9.8 | n/a | Pre PCV | Non-bacteraemic:  PCV7: 51/161 (31.7)  PCV10: 64/161 (39.8)  PCV13: 98/161 (60.9)  PPV23:121/161 (75.2)  Bacteraemic:  PCV7: 19/94 (20.2)  PCV10: 28/94 (40.4)  PCV13: 66/94 (70.2)  PPV23: 80/94 (85.1) | i |
| Harat 2016 | Poland | P, surveillance | 2010-2012 | Culture (blood, acceptable quality respiratory) or BinaxNOW or UAD | ≥50:  50-64 (52.5%); 65-69 (12.8%); 70-79 (25.0%); 80+ (9.3%) | 144/1195 (12.1) | Pre PCV (no national immunisation efforts although PCV available) | PCV13: 76/77 (98.7) | a,b,d,e |
| Horacio 2018 | Portugal | R, surveillance | 2012-2015 | Cultures from sputum, bronchial secretions, BAL. Capsular reaction test using chessboard system and specific antisera | ≥50:  50-64 (26.6%); ≥65 (49.8%) | n/a | 2-5 | **≥50:**  PCV13: 361/1096 (32.9)  PPV23 642/1096 (58.6)  ≥65:  PCV13: 242/714 (33.9)  PPV23: 422/714 (59.1) | **f,g,h,i** |
| Horacio 2014 | Portugal | R, lab-based surveillance | 1999-2011 | Cultures from sputum, bronchial secretions, BAL. Capsular reaction test using chessboard system and specific antisera | ≥50:  50-64 (27.3%); ≥65 (47.7%) | n/a | Pre to 1 year post | **≥50:**  PCV13: 97/225 (43.1)  PPV23: 153/225 (68.0)  **≥65:**  PCV13: 58/143 (40.6)  PPV23: 96/143 (67.1) | **No** |
| Isturiz 2019 | USA | P, surveillance | 2013-2016 | SPn isolated or BinaxNow or UAD | ≥65 | 585/6347 (9.2) | 3-5 | PCV13: 265/585 (45.3) | a,b,c,d,e,f,g,h |
| Kim 2019 | Korea; China; Philippines; Singapore; Thailand | P, surveillance | 2012-2017 | Culture – blood, LRT specimens, pleural fluid; Quellung method with commercial antisera | Mean 68.6 (10.6)  ≥50 | n/a | Korea: 2-5;  China: pre;  Philippines: 2-5;  Singapore: 1-4;  Thailand: 1-4 | PCV13:  Korea: 54/146 (37.0); China: 105/194 (54.1);  Philippines: 17/49 (34.7);  Singapore: 1/4 (25);  Thailand: 35/55 (63.6)  PPV23:  Korea: 81/146 (55.5); China: 108/194 (55.7);  Philippines: 28/49 (57.1);  Singapore: 2/4 (50);  Thailand: 41/55 (74.5) | f,g,h,i |
| LeBlanc 2020 | Canada | P, surveillance | 2010-2015 | Blood/sputum culture or urinary BinaxNOW or ssUAD (13-valent) | ≥50 (65.5%); ≥65 (46.2%) | 647/4723 (11.3) | 1-5 | PCV13:  ≥50; 311/647 (48.1)  ≥65: 173/391 (44.2) | a,b,c,d,e,f,,h |
| LeBlanc 2019 | Canada | P, surveillance | 2010-2015 | Blood/sputum culture or urinary BinaxNOW or ssUAD (13-valent) | ≥50; ≥65 | See LeBlanc 2020 |  | PCV13:  Non-bacteraemic:  201/3534 (5.7% all CAP with negative BC)  Bacteraemic:  370/5405 (6.8) | No |
| Menendez 2017 | Spain | PC | 2011-2014 | Blood/sputum/pleural culture isolate or BinaxNOW or ssUAD | 67.4 ±17.9 | 368/1258 (29.3) | 1-3 | PCV13: 221/368 (60.1)  PCV7: 44/368 (12.0) | a,b,d,d,e,f,g,h |
| Morimoto 2015 | Japan | P surveillance | September 2011-January 2013 | Blood/sputum culture, multiplex PCR on sputum, BinaxNOW. Quellung reaction. | 50-64 (14%); 65-74 (18%); 75-84 32%); 85+ (25%) | 26.2% (whole cohort) | n/a | Data no age- specific  Incidence data for SPn CAP. | No |
| Noguchi 2019 | Japan | PC | 2011-2017 | Culture (sputum, intratracheal, BAL). Quellung reaction | Mean 67.0 ±13.6 | n/a | Up to 4 years | PCV13:101/229 (44.1)  PPV23: 140/229 (61.1) | f,g,h,i |
| Oishi 2006 | Japan | PC | 2001-2003 | Culture (blood, LRT) | Mean 67.4 ±15.8 | n/a | Pre PCV | PPV23: 94/114 (82.5) | i |
| Pick 2020 | UK | PC | 2013-2018 | Blood culture or pneumococcal UAD or ssUAD (24 valent) | 50-64 (20.1%); 65-74 20.8%); 75-84 25.8%); 85+ (15.5%) | ≥50:  864/2901 (29.8) | 3-8 | ≥50:  PCV13: 285/781 (36.5)  PPV23: 583/837 (69.6) | a,b,c,d,e,f,g,h,i |
| Pletz 2016 | Germany | RC | October 2002-December 2011 | Selected population to compare serotype distribution – culture negative but urinary BinaxNOW positive | 63.0 ±17.4 (2002-06);  57.5 ±16.1 (2007-11) | n/a | Pre- and up to 4 years post PCV7;  Pre PCV 13 | **Period 1 (pre PCV7):**  PCV7: 60/196 (30.6)  PCV13: 123/196 (62.8)  **Period 2 (post PCV7):**  PCV7: 26/195 (13.3)  PCV13: 120/195 (61.5) | **No** |
| Prato 2018 | Italy | P, population-based, surveillance | January 2013-January 2015 | Blood, NP swabs, BAL, sputum. PCR and multiplex sequential PCR | ≥65 (Median 79 (IQR 73-85)) | 59/186 (31.7) | 3-5 | PCV13: 39/59 (66.1)  PPV23: 48/59 (81.4) | a,b,c,d,e,f,g,h,i |
| Quirk 2019 | Iceland | Lab-based cohort | PrePCV10: 2009-11  Post PCV10 I: 2012-14  Post PCV10 II: 2015-17 | Pneumococcal isolates from LRT. Serotyped using ImmuLex pool antisera and/or multiplex PCR | Median 75.2  ≥65 | n/a | Up to 6 years post | ≥65: PCV10:  Pre-vac 107/191 (56.0)  Post-vac I (1-3 years post PCV) 65/137 (47.4)  Post vac II (4-6 years post PCV)  19/102 (18.6) | No |
| Regev-Yochay 2018 | Israel | PC | March 2014-July 2015 | Culture (blood/pleural fluid/BAL/tracheal aspirate, respiratory)/BinaxNOW/ss-UAD (13-valent) | Mean 72.7 ±10.9  50-<65 (26.9%); 65-<80 (45.2%); 80+ (27.9%) | 80/498 (16.1) | 4 | ≥50:  PCV13: 38/80 (47.5) | a,b,c,d,e,f,g,h |
| Rodrigo 2015 | UK | PC | 2008-2013 | Blood culture/urinary BinaxNOW/ss-UAD | 45-64 (22.2%); 65-74 (20.8%); 75-84 (25.4%); 85+ (16.2%) | 87/653 (13.3) | Up to 3 years post | ≥45:  Post PCV13: 112/244 (45.9)  ≥65:  Post PCV13:  76/177 (42.9) | a,b,c,d,e,f,g,h |
| Sando 2019 | Japan | PC | 2011-2017 (May 2016-April 2017 = Post phase 2) | Pneumococcal isolates from blood, sputum, or pleural fluid. Quellung method. | Median 73 (IQR 63-83)  ≥65= 72% | n/a | Up to 3 years | Post PCV13:  71/215 (53) | f,g,h,i |
| Sherwin 2013 | USA | P cross-sectional | February 2010 to September 2011 | Blood/respiratory culture/BinaxNow. Ss-UAD or Quellung reaction | Mean 65.4 (range 50-102) | 98/710(13.8) | 1 | PCV13:  78/98 (79.6)PC | a,b,c,d,e,f,g,h |
| Suzuki 2017 | Japan | PC | September 2011 to August 2014 | Blood/sputum culture. Quellung reaction on isolates. Sputum PCR for pneumolysin and autolysin genes, nanofluidic real-time PCR for 50 STs if positive. Urinary BinaxNOW. | ≥65 | 419/2036 (20.6) | Up to 1 year post PCV13 | PCV13:202/344 (58.7)  PPV23: 272/344 (79.1) | a,b,d,e,i |
| van Werkhoven 2016 | Netherlands | PC | 2008-2013 | Culture from normally sterile respiratory tract specimen/BinaxNOW/SS-UAD (13-valent). Isolates – Quellung reaction | ≥65 | - | Up to 2 years post PCV10 | 1-2 years post PCV10:  PCV10: 19/60 (31.7)  PCV13: 34/60 (56.7) | f,g,h |
| Vestjens 2017 | Netherlands | RC | 2004-2016 | Blood culture, sputum culture, BinaxNOW. ST-specific 25-plex pneumococcal serum antibody assay. Isolates -Quellung reaction | Mean age (post PCV10) 64.4 ±16 | 155/505 (30.7) | Post PCV10: 1-5 | Post PCV10:  10/77 (13.0) | a,b,c,d,e |
| Vila-Corcoles 2016 | Spain | PC | December 2008 to December 2011 | Blood, sputum culture, BinaxNOW. ST at reference lab (?method) | ≥60:  60-99 (45.6%); 70-79 (34.1%); 80+ (20.3%) | n/a | Pre-PCV | PCV13:15/28 (53.6)  PPV23: 19/28 (67.8) | i |
| Wunderink 2018 | USA | RC | 2010-2012 | Culture (blood, good quality sputum, endotracheal aspirate, BAL, pleural fluid), BinaxNOW, ss-UAD (13 valent). Isolates serotyped by Quellung reaction or PCR. Ss-UAD in selected group from EPIC study | Mean age 50+ | 169/1736 (9.7) | Up to 2 years | PCV13: 110/169 (65.1) | a,b,c,d,e,f,g,h |

Abbreviations:

BAL – broncho-alveolar lavage

| Meta-analyses | |
| --- | --- |
| a | Proportion CAP due to S.pneumoniae pre and post PCV10/13 introduction |
| b | Proportion CAP due to S.pneumoniae stratified by serotyping method |
| c | Proportion CAP due to S.pneumoniae stratified by continent |
| d | Proportion of CAP due to S.pneumoniae by definition of age |
| e | Proportion of CAP due to S.pneumoniae by depth testing |
| f | Proportion of pneumococcal CAP due to PCV13 VT by serotyping method |
| g | Proportion of pneumococcal CAP due to PCV13 VT post PCV10/13 introduction |
| h | Proportion of pneumococcal CAP due to PCV13 VT by continent |
| i | Proportion of pneumococcal CAP due to PPV23 VT |

CAP – community-acquired pneumonia

IQR – interquartile range

LRT – lower respiratory tract

MA – meta-analysis

P - prospective

R - retrospective

PC - Prospective cohort

RC - Retrospective cohort

Spn – *Streptococcus pneumoniae*

ssUAD – serotype-specific urinary antigen detection

VT – vaccine type

## Supplemental Table 2: Modified Newcastle-Ottawa Scale risk of bias assessment

| **Study** | **Selection domain (max 2)** | **Outcome domain (max 1)** | **Total** |
| --- | --- | --- | --- |
| Benfield 2013 | 1 | 1 | 2 |
| Choi 2015 | 2 | 1 | 3 |
| Di Pasquale 2017 | 1 | 1 | 2 |
| Domenech 2011 | 1 | 1 | 2 |
| Harat 2016 | 2 | 1 | 3 |
| Horacio 2018 | 1 | 1 | 2 |
| Horacio 2014 | 1 | 1 | 2 |
| Isturiz 2019 | 2 | 1 | 3 |
| Kim 2019 | 1 | 1 | 2 |
| LeBlanc 2020 | 2 | 1 | 3 |
| LeBlanc 2019 | 2 | 1 | 3 |
| Menendez 2017 | 2 | 1 | 3 |
| Morimoto 2015 | 2 | 1 | 3 |
| Noguchi 2019 | 2 | 1 | 3 |
| Oishi 2006 | 2 | 1 | 3 |
| Pick 2020 | 2 | 1 | 3 |
| Pletz 2016 | 1 | 1 | 2 |
| Prato 2018 | 2 | 1 | 3 |
| Quirk 2019 | 0 | 1 | 1 |
| Regev-Yochay 2018 | 2 | 1 | 3 |
| Rodrigo 2015 | 2 | 1 | 3 |
| Sando 2019 | 1 | 1 | 2 |
| Sherwin 2013 | 2 | 1 | 3 |
| Suzuki 2017 | 2 | 1 | 3 |
| van Werkhoven 2016 | 2 | 1 | 3 |
| Vestjens 2017 | 2 | 1 | 3 |
| Vila-Corcoles 2016 | 2 | 1 | 3 |
| Wunderink 2018 | 2 | 1 | 3 |

## Supplementary Figure 1: Estimated proportion of CAP due to *S.pneumoniae* in the periods prior to and post introduction of PCV10/13 immunisation in children

Heterogeneity between groups: p = 0.417

Overall

(I^2 = 98.67%, p <0.01);

**Pre PCV10/13**

Choi (2011-13)

Suzuki (2011-14)

Subtotal

(I^2 = 98.94%, p <0.01)

Isturiz (2013-15)

DiPasquale (2011-12)

Choi (2007-10)

Prato (2013-15)

Pick (2013-18)

Menendez (2011-14)

Regev (2014-15)

Vestjens (2012-16)

Subtotal

(I^2 = 97.09%, p <0.01)

Wunderink (2010-12)

Vestjens (2004-11)

**Post PCV10/13**

Rodrigo (2010-13)

Rodrigo (2008-09)

Sherwin (2010-11)

Study

Harat (2010-12)

LeBlanc (2010-15)

100.00

5.93

6.01

64.93

6.05

5.51

5.93

5.49

6.03

5.98

5.84

5.70

35.07

%

6.00

5.84

5.96

5.81

5.91

5.97

6.05

0.19 (0.15, 0.24)

0.11 (0.09, 0.13)

0.21 (0.19, 0.22)

0.18 (0.13, 0.24)

0.09 (0.09, 0.10)

0.18 (0.13, 0.24)

0.15 (0.13, 0.18)

0.32 (0.25, 0.39)

0.30 (0.28, 0.31)

0.29 (0.27, 0.32)

0.16 (0.13, 0.20)

0.26 (0.21, 0.31)

0.22 (0.15, 0.29)

0.10 (0.08, 0.11)

0.31 (0.27, 0.35)

0.23 (0.20, 0.25)

0.14 (0.11, 0.17)

ES (95% CI)

0.12 (0.10, 0.14)

0.11 (0.11, 0.12)

100.00

5.93

6.01

64.93

6.05

5.51

5.93

5.49

6.03

5.98

5.84

5.70

35.07

%

6.00

5.84

5.96

5.81

5.91

Weight

5.97

6.05

.25

.5

.75

1

Proportion

0.38 (0.34, 0.43)

Sensitivity analysis excluding 1 study (di Pasquale) considered to be at some risk of selection bias:

Pre PCV10/13 : 23% (16-29)

Post PCV10/13: 18% (15-24)

Overall I^2^=98.75%

## Supplemental Figure 2 : Estimated proportion of CAP due to *S.pneumoniae* stratified by definition of age

Heterogeneity between groups: p = 0.641

Overall (I^2 = 98.67%, p <0.01));

Rodrigo

Pick

Vestjens

Study

Subtotal (I^2 = 99.08%, p <0.01)

Suzuki

Isturiz

Harat

Regev

LeBlanc

Choi

Prato

**No**

Subtotal (I^2 = 97.70%, p <0.01)

Choi

DiPasquale

Rodrigo

Vestjens

Menendez

Wunderink

Sherwin

**Yes**

2008-09

2013-18

2004-11

period

2011-14

2013-15

2010-12

2014-15

2010-15

2011-13

2013-15

2007-10

2011-12

2010-13

2012-16

2011-14

Study

2010-12

2010-11

100.00

5.81

6.03

5.84

Weight

53.62

6.01

6.05

5.97

5.84

6.05

5.93

5.49

46.38

5.93

5.51

5.96

5.70

5.98

%

6.00

5.91

0.19 (0.15, 0.24)

0.38 (0.34, 0.43)

0.30 (0.28, 0.31)

0.31 (0.27, 0.35)

ES (95% CI)

0.19 (0.13, 0.25)

0.21 (0.19, 0.22)

0.09 (0.09, 0.10)

0.12 (0.10, 0.14)

0.16 (0.13, 0.20)

0.11 (0.11, 0.12)

0.11 (0.09, 0.13)

0.32 (0.25, 0.39)

0.21 (0.14, 0.28)

0.15 (0.13, 0.18)

0.18 (0.13, 0.24)

0.23 (0.20, 0.25)

0.26 (0.21, 0.31)

0.29 (0.27, 0.32)

0.10 (0.08, 0.11)

0.14 (0.11, 0.17)

100.00

5.81

6.03

5.84

Weight

53.62

6.01

6.05

5.97

5.84

6.05

5.93

5.49

46.38

5.93

5.51

5.96

5.70

5.98

%

6.00

5.91

.25

.5

.75

Proportion

Yes= defined age strata

No = mean or median age

Sensitivity analysis excluding 1 study (di Pasquale) considered to be at risk of some selection bias:

Yes : 19% (13-25)

No: 21% (14-29)

Overall I^2^=98.75%

## Supplemental Figure 3: Proportion of CAP due to *S.pneumoniae* stratified by depth of testing

##

Heterogeneity between groups: p = 0.711

Overall

(I^2 = 98.67%, p < 0.01);

Prato

Subtotal

(I^2 = 93.27%, p < 0.01)

Wunderink

Harat

Choi

LeBlanc

Rodrigo

Isturiz

Vestjens

Regev

Study

Subtotal

(I^2 = 98.96%, p < 0.01)

Menendez

Vestjens

DiPasquale

Choi

Rodrigo

Pick

**sub-optima**l

**optimal**

Suzuki

Sherwin

2013-15

2010-12

2010-12

2011-13

Study

2010-15

2010-13

2013-15

2004-11

2014-15

period

2011-14

2012-16

2011-12

2007-10

2008-09

2013-18

2011-14

2010-11

5.97

%

6.05

5.84

Weight

5.51

5.81

6.03

6.01

0.19 (0.15, 0.24)

0.32 (0.25, 0.39)

0.18 (0.12, 0.26)

0.10 (0.08, 0.11)

0.12 (0.10, 0.14)

0.11 (0.09, 0.13)

0.11 (0.11, 0.12)

0.23 (0.20, 0.25)

0.09 (0.09, 0.10)

0.31 (0.27, 0.35)

0.16 (0.13, 0.20)

ES (95% CI)

0.20 (0.15, 0.25)

0.29 (0.27, 0.32)

0.26 (0.21, 0.31)

0.18 (0.13, 0.24)

0.15 (0.13, 0.18)

0.38 (0.34, 0.43)

0.30 (0.28, 0.31)

0.21 (0.19, 0.22)

0.14 (0.11, 0.17)

100.00

5.49

22.86

6.00

5.93

5.96

6.05

5.84

77.14

5.98

5.70

5.93

5.91

.25

.5

.75

Proportion

Sensitivity analysis excluding 1 study (di Pasquale) considered to be at some risk of selection bias:

Optimal : 20% (15-25)

Suboptimal: 18% (10-28)

Overall I^2^=98.75

## Supplemental Figure 4: Estimated proportion of pneumococcal CAP caused by PCV13 vaccine type *S. pneumoniae* in the period after the introduction of PCV 10/13 programmes


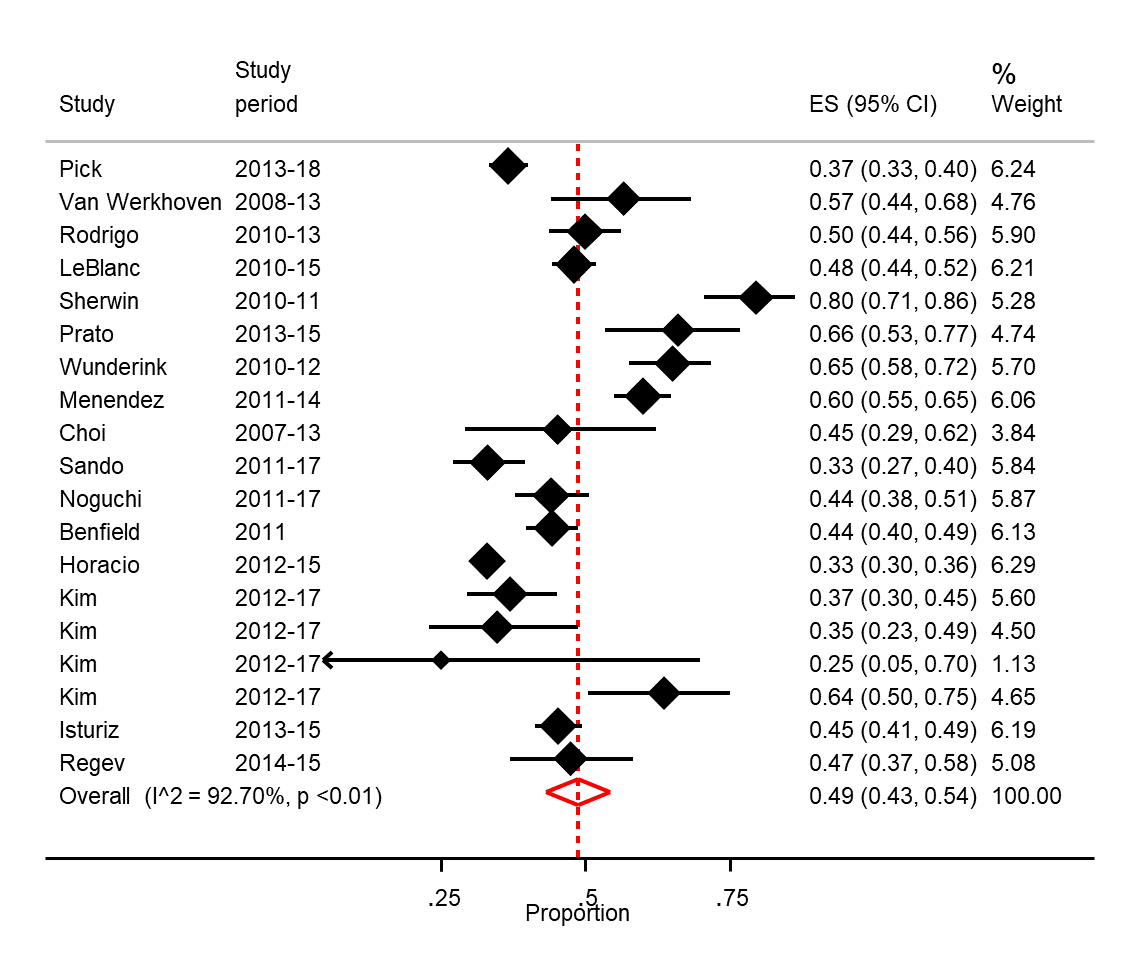


Sensitivity analysis excluding 4 studies (Benfield, Horacio, Kim, Sando) considered to be at some risk of selection bias:

Estimated overall proportion: 54% (47-60); I^2^=92.14%

## Supplemental Figure 5: Estimated proportion of pneumococcal CAP due to PCV13 vaccine serotypes – stratified by continent.

Heterogeneity between groups: p = 0.057

Overall (I^2 = 92.70%, p <0.01);

Wunderink

N America

Subtotal (I^2 = 94.31%, p <0.01)

Isturiz

Europe

Menendez

Regev

Benfield

Sherwin

Sando

LeBlanc

Pick

Study

Kim

Asia

Prato

Subtotal (I^2 = 70.09%, p<0.01)

Noguchi

Rodrigo

Kim

Subtotal (I^2 = 94.98%, p <0.01)

Choi

Horacio

Kim

Van Werkhoven

Kim

USA

USA

Spain

Israel

Denmark

USA

Japan

Canada

UK

Country

Korea

Italy

Japan

UK

Philippines

Korea

Portugal

Singapore

Netherlands

Thailand

2010-12

2013-15

2011-14

2014-15

2011

2010-11

2011-17

2010-15

2013-18

period

2012-17

2013-15

2011-17

2010-13

2012-17

2007-13

2012-15

2012-17

2008-13

2012-17

Study

0.49 (0.43, 0.54)

0.65 (0.58, 0.72)

0.48 (0.40, 0.56)

0.45 (0.41, 0.49)

0.60 (0.55, 0.65)

0.47 (0.37, 0.58)

0.44 (0.40, 0.49)

0.80 (0.71, 0.86)

0.33 (0.27, 0.40)

0.48 (0.44, 0.52)

0.37 (0.33, 0.40)

ES (95% CI)

0.37 (0.30, 0.45)

0.66 (0.53, 0.77)

0.41 (0.34, 0.49)

0.44 (0.38, 0.51)

0.50 (0.44, 0.56)

0.35 (0.23, 0.49)

0.59 (0.47, 0.71)

0.45 (0.29, 0.62)

0.33 (0.30, 0.36)

0.25 (0.05, 0.70)

0.57 (0.44, 0.68)

0.64 (0.50, 0.75)

100.00

5.70

45.19

6.19

6.06

5.08

6.13

5.28

5.84

6.21

6.24

Weight

5.60

4.74

31.43

5.87

5.90

4.50

23.37

3.84

6.29

1.13

4.76

4.65

%

0.49 (0.43, 0.54)

0.65 (0.58, 0.72)

0.48 (0.40, 0.56)

0.45 (0.41, 0.49)

0.60 (0.55, 0.65)

0.47 (0.37, 0.58)

0.44 (0.40, 0.49)

0.80 (0.71, 0.86)

0.33 (0.27, 0.40)

0.48 (0.44, 0.52)

0.37 (0.33, 0.40)

ES (95% CI)

0.37 (0.30, 0.45)

0.66 (0.53, 0.77)

0.41 (0.34, 0.49)

0.44 (0.38, 0.51)

0.50 (0.44, 0.56)

0.35 (0.23, 0.49)

0.59 (0.47, 0.71)

0.45 (0.29, 0.62)

0.33 (0.30, 0.36)

0.25 (0.05, 0.70)

0.57 (0.44, 0.68)

0.64 (0.50, 0.75)

100.00

5.70

45.19

6.19

6.06

5.08

6.13

5.28

5.84

6.21

6.24

Weight

5.60

4.74

31.43

5.87

5.90

4.50

23.37

3.84

6.29

1.13

4.76

4.65

%

.25

.5

.75

Proportion

Sensitivity analysis excluding 4 studies (Benfield, Horacio, Kim, Sando) considered to be at some risk of selection bias:

Europe: 52% (42-63)

North America: 59% (47-71)

Asia: 44% (38-50)

Overall I2=92.14%

## Supplemental Figure 6: Estimated proportion of pneumococcal CAP due to PPV23 vaccine type stratified by serological test method

7.15

%

1.23

13.21

5.68

0.67 (0.62, 0.72)

0.55 (0.47, 0.63)

0.80 (0.75, 0.85)

0.65 (0.58, 0.71)

0.79 (0.74, 0.83)

0.70 (0.66, 0.73)

ES (95% CI)

0.55 (0.38, 0.71)

0.68 (0.49, 0.82)

0.70 (0.66, 0.74)

0.51 (0.44, 0.57)

0.82 (0.74, 0.88)

0.57 (0.43, 0.70)

0.61 (0.55, 0.67)

0.75 (0.62, 0.84)

0.59 (0.56, 0.61)

0.56 (0.49, 0.62)

0.50 (0.15, 0.85)

0.80 (0.75, 0.83)

0.81 (0.70, 0.89)

100.00

7.27

78.95

7.52

7.84

Weight

4.50

4.30

7.66

7.23

6.62

5.36

7.28

5.56

7.89

%

Heterogeneity between groups: p <0.001

Overall (I^2 = 90.31%, p <0.01);

Kim

Domenech

Subtotal (I^2 = 88.16%, p <0.01)

UAD24

Suzuki

Pick

Study

Isolates

Choi

Vila-Corcoles

Benfield

Sando

Oishi

Kim

Noguchi

PCR

Kim

Horacio

Kim

Kim

Subtotal (I^2 = .%, p = .)

Prato

2012-17

2001-08

2011-14

2013-18

period

2007-13

2008-11

2011

2011-17

2001-03

2012-17

2011-17

2012-17

2012-15

2012-17

Study

2012-17

2013-15

0.67 (0.62, 0.72)

0.55 (0.47, 0.63)

0.80 (0.75, 0.85)

0.65 (0.58, 0.71)

0.79 (0.74, 0.83)

0.70 (0.66, 0.73)

ES (95% CI)

0.55 (0.38, 0.71)

0.68 (0.49, 0.82)

0.70 (0.66, 0.74)

0.51 (0.44, 0.57)

0.82 (0.74, 0.88)

0.57 (0.43, 0.70)

0.61 (0.55, 0.67)

0.75 (0.62, 0.84)

0.59 (0.56, 0.61)

0.56 (0.49, 0.62)

0.50 (0.15, 0.85)

0.80 (0.75, 0.83)

0.81 (0.70, 0.89)

100.00

6.89

7.27

78.95

7.52

7.84

Weight

4.50

4.30

7.66

7.23

6.62

5.36

7.28

5.56

7.89

6.89

7.15

1.23

13.21

5.68

.75

.5

.25

Proportion

Note Suzuki: induced sputum obtained in non-productive patients – examined for 50 STs by real-time PCR if positive for both pneumolysin and autolysin by PCR

Sensitivity analysis excluding 4 studies (Benfield, Horacio, Kim, Sando) considered to be at some risk of selection bias:

PCR: 80% (75-83)

Isolates: 68% (53-81)

Overall I^2^=83.97%
